# Dentistry as a Fine Art. No. 4

**Published:** 1867-09

**Authors:** Norman W. Kingsley

**Affiliations:** Professor of Dental Art and Mechanism, in the New York College of Dentistry.


(To be Continued.)

---

### ARTICLE V.

### *Dentistry as a Fine Art. No. 4.*

By Norman W. Kingsley, Professor of Dental Art and Mechanism, in the New York College of Dentistry.

In our last article we presented a type of natural teeth, offering it as a standard for comparison.

It is not to be expected that the artist, be he manufacturer or dentist will conform to it strictly to any great extent. The instances in which one peculiar form is the very best that could be selected as adapted to all the requirements of the case, are few, compared with the whole number. In the type presented we find a beauty of form that is rarely seen except in youth.

The undulation of the cutting edges of the incisor, in the friction of antagonism, soon give way, to a line more nearly square with the sides of the tooth.

Any one of but limited observation has noticed, in many cases, the serrated edges of the incisors, both superior and inferior, immediately after eruption, and also that in a little time this peculiarity has passed away.

This wearing away of the antagonizing ends of the teeth is the most natural modification of the perfect form of the tooth, and is common to them all.

A great variety of forms can be made, all harmonizing with what we see in nature by taking a well developed type, and producing the appearances above indicated.

Thus, by cutting off the ends of the teeth as exhibited in the illustration, we give the semblance of age, and that without in the least changing the form of the upper portion.

By having the mind clearly impressed with an ideal standard, appropriate selections from a ready-made stock will be more easily made; or when the desired form is not supplied, changes may be secured to a limited extent by grinding. One thing is to be especially avoided; mannerism. The adoption in all cases of any type, or its variations, however excellent, can only end in deformity.

Too many artists are mere mannerists, either by carrying some single idea of their own into all their works, or what is more common, copying the modes and peculiarities of genius, and thus caricaturing, rather than imitating nature. Mannerism is always an evidence of weakness.

For a complete knowledge of probable and possible variations, the student must be a close observer of nature. His standard of beauty will finally be the result of the rejection of nature's defects, and the combination of her excellence. Imitate nature, rather than attempt to copy her. A copy of any one presentation would not probably convey as pleasing an impression as an adaptation of an imitation by an artist who had throughly studied the requirements of the case.

That method of making artificial teeth which requires, for success, the possession of the highest order of artistic talent, is undoubtedly carving.

In these cases the artist cannot to any extent copy nature; he is compelled to imitate her, and upon that art which con-conceals art his success depends. Not only must he carve each individual member with a character which shall harmonize with the external features, but the arrangement or

("grouping," as an artist would term it,) must be the result of most careful study. There is another style of work which requires a lower order of talent, but in which the results are in many cases quite equal to the best efforts at carving. Continuous Gum work, known as the invention of Dr. John Allen, is the result of the arrangement of single teeth in any desired form, and the completion of the operation by forming around them an artificial gum.

No doubt, if the teeth in the market were in form, color and variety, all that is needed to meet the requirements, this method of forming an artificial denture would be all that art demands. It would then possess all the merit of carved work, and in some respects afford even greater opportunity for artistic display; being also much easier of accomplishment. As it is, the same taste and study are required in grouping, as in carved blocks.

Absolute rules cannot be given for this art. Suggestions only can be made which may prove a valuable aid. It must be borne in mind that we are not dealing with the natural organs, and some allowances and deviations must be made for that almost imperceptible difference in appearance that exists in the artificial ones even when they are the most perfect of their class.

Well-formed natural teeth please the eye when symmetrically placed even close together in the arch.

Artificial teeth under like arrangement nearly always betray their origin.

In this connection we may gather much profit from the directions given to the painter. The general instructions are pertinent and applicable.

"Nothing is so injurious to effect as a crowded picture. If the subject requires the introduction of many figures they should be distributed in masses, or groups, in different gradations. The object in breaking a composition into groups is that the eye in passing from one to another, may, by having a distinct classification of the parts, easily comprehend the whole. Figures should be more or less varied in attitude, because an exact repetition of line produces formality. The manner and extent of variation must be decided by the subject. They must also vary in regard to prominence. The artist who represents all the figures introduced in his picture as holding the same rank, making each one equally prominent, understands nothing of the principles of nature, or the laws of art. The same artist will, with great labor, bring forward on his canvas, the most insignificant objects; for trivial minds ever value trivial things."

"Nature never repeats herself, even in two sides of a leaf."

"Such precision belongs to machine work; and, in studying nature we learn that variety is no less necessary to a pleasing composition than unity. To the grace and beauty of the whole work, harmony is indispensible. Without harmony each part may fail of the effect intended, however true in design. There must be harmony of line, harmony of grouping, harmony of light and shade, harmony of coloring, harmony of expression; each part must be so adapted as to correspond to the rest. The attitude must be in keeping with the expression; the color, with the subject treated; and the accessories must be true, both to the character and the age represented: a harmonious whole is always more or less pleasing in itself, independent of subject or style."

"Unity is distinct from harmony, and requires one point of view, one focus of light, one tone of color. There may be unity of parts when harmony in the whole is entirely wanting. In learning the rules for composition, as in all other departments of art, the artist must study nature to find his fundamental principle."

"We find every degree of strength and beauty, every variety of element and every possible variety of combination in the human form and character, and according to the law of harmony that pervades life, we also find, that the intermediate combination, that serves to unite and harmonize the two extremes, partaking alike of the character of both, is never wanting.''

The application of these principles for a number of years in the arrangement of artificial teeth, has satisfied the writer that in no other way can so pleasing effects be produced.

The gratification of the eye by a judicious deviation from uniformity is nowhere more strikingly illustrated than in landscape gardening. The traveler who is familiar with the ancient parks or gardens of the continent of Europe, laid out with all the regularity of squares on a chess board ; the trees and shrubbery often trimmed or twisted into fantastic shapes unlike the free growth of nature—experiences a sense of great relief, in visiting the parks of England where the art in the arrangement is less mechanical and more concealed.

This formality and stiffness is not displeasing at first, to the uncultivated, but the eye soon wearies of it and seeks relief in variety. It is this action of the mind we must consult in the arrangement of artificial teeth ; and in doing so, it does not follow that the mind will be able to recognize the cause of that which gratifies it. The æsthetic sense may be fully satisfied without being aware of the true reasons of the satisfaction.

We have shown in a former article the undulations of line manifest in every view of each tooth. To harmonize with this character, the arrangement of the whole must avoid straight lines. The teeth ought to be so placed that their cutting or grinding ends will not all be upon the same level.

Borrowing again from the art of drawing in perspective ; we must first establish the horizon line ; and our deviations will be made from that. Thus, a very natural order is, to bring the cutting edges of the incisors down to the horizon line ; let the lateral incisor stop short, and carry the point of the canine below the line. Let the bicuspids touch the line, place the first molar above it, the second molar higher still ; and the third molar higher than either.

Any material deviation from such an arrangement will nearly always incline to deformity.

For the position in the arch, an almost indefinite variety is admissable ; comprehending their rotation on their axes, the spaces which separate them and deviations from a uniform curve. Artificial teeth will always look better if placed so that the form of each is clearly relieved against the shadow of the spaces between them.

A compact row in an aged person where there are many external evidences that nature is giving way, strikes almost any one as an incongruity. With age we see a shifting of position of the natural teeth which is in perfect harmony with the wasting of muscle and tissue which characterizes advanced life.

For the best artistic effect, the spaces between the teeth should not be uniform in width, but there may be comparative uniformity in the two sides of the mouth.

In general the central incisors will look better if nearly or quite in contact, but may be relieved by a space between them and the laterals. The canine may, or may not, be separated from the lateral by a decided space, but a considerable vacancy may be left between the canine and bicuspid without any detriment to beauty.

In addition to the variety that may be caused by a judicious distribution of spaces, a most wonderful effect upon the expression will be caused by the partial rotation of a tooth, or by giving prominence to one or more in the arch.

For natural expression, the central incisors and canines must occupy a fixed and unalterable location.

The centrals control the *profile* of the mouth, while the canines support and give character to its *corner* ; the two most important points in the whole arrangement.

There can be but little or no deviation admissable in the location of these teeth, but a partial twisting or a different inclination may often be resorted to with good effect.

The central incisors will however generally appear better by having them stand with their flat faces on a line with each other, but the canines may be rotated slightly, in either the same or opposite directions without disfigurement.

The lateral incisors and bicuspids should occupy positions subordinate to the canines and centrals. Considerable latitude may be shown in placing the lateral. It may have a greater inclination toward the median line than the others, or it may be twisted more on its axis ; the anterior corner of the cutting edge may be thrown forward of the central, or the whole tooth may stand within the arch, either method carried to a limited extent will add to the naturalness of the effect.

The bicuspids should stand within a curved line formed by the centrals, canines, and molars, partially hidden by the prominence of the canine.

The foregoing remarks apply entirely to the construction of an upper denture, but are of greater force when an entire upper and lower denture are to be supplied.

The character decided upon for the upper, will govern the character to be given to the lower set.

Where any great number of the natural teeth remain upon the lower jaw and an entire upper set is to be supplied, the character of the lower teeth will influence the form and arrangement of the artificial ones and thus the suggestions before made will be modified to meet the case.

Perfect harmony would therefore require that noticeable defects or irregularities in the lower natural teeth should be imitated in a modified form, in the construction of the upper.

Thus, marked irregularities of position, below, will indicate an irregular arrangement above, but not necessarily to the same extent.

Permanent discolorations on the surface of the natural teeth would also indicate a modified imitation of the same on the artificial.*

It is the comparative perfection of artificial teeth, together with their stiffness and formality, which, even if the color be appropriate, betrays them in persons of full age.

Tricks or devices may be justifiably resorted to in such cases. The grinding of the cutting edges to produce the appearance of a natural tooth broken or bruised by abrasion, is such a device, and may be adopted occasionally with much benefit. Not that there is any intrinsic beauty in a broken tooth ; nor that there is any charm in its contrast with a perfect one, but the eye is so accustomed to see these slight defects in the natural teeth, that it comes to regard them as only allied to nature.

The insertion of gold fillings in exposed portions of the teeth is another trick which can sometimes be made available with propriety. In the construction of a partial set where there are fillings in the natural teeth which are exposed to the ordinary observer; harmony demands that there be no large number of artificial teeth inserted, perfect in their form and appearance. It is then eminently proper to adopt this device, but the filling should not be conspicuous or obtrusive. In making an entire set this trick has very little to recommend it. The means at our command in such cases are sufficient to enable us to conceal our art without resorting to the questionable device of suggesting to the mind, decay, and thus induce the inference

---

*In 1852 I was called upon to insert an upper set of teeth where the natural lower teeth were sound, but stained by long neglect, in marked and irregular spots over the surface.

After finding myself unable to remove the discolorations, I resolved to imitate them, and carved a set of teeth and stained them in the baking with a preparation of *terra di sienne*. After they had been worn a year they were exhibited in the mouth of the patient to the Jurors at the "World's Fair" in New York in 1853, and elicited their highest commendation. Vide Jurors' report.

that the organs are natural. In the case of the partial set, harmony with the exposed natural teeth demands it, but in an entire set it is of doubtful propriety.

But few words can be added to the remarks in a former article on color. The canine teeth in nature are less translucent and more deeply shaded, than the incisiors or bicuspids. This should certainly be imitated so far as the canines are concerned, but in the opinion of the writer, we shall produce a better effect with artificial teeth by *not* inserting bicuspids of a lighter shade than the canines.

The artificial tooth does not absorb the light as does the natural one, and when placed in shadow as the bicuspids *in situ*, they are rendered more conspicuous. Where natural teeth of divers colors are scattered, and the vacancies are to be supplied, it is our duty to harmonize in color each artificial tooth with its natural neighbor.*

It will be manifest that it is simply impossible to carry all the foregoing suggestions into practice with some of the methods of constructing sets of teeth now in use.

With gum teeth for plate work there is but little latitude for artistic effect consistent with the mechanical execution ; with blocks made for rubber there is still less freedom of arrangement for the operator.

Single teeth without gums are the only ones as a general rule, that will permit us to exercise our taste unlimitedly.

In entire sets where the absorption of the alveolar process necessitates a substitute to restore the contour, we find

---

* I was required on one occasion to insert the four superior incisors. One of the canines was of exceedingly fair color, the other was very much discolored by a black amalgam filling on its anterior approximal surface, which the patient on no account would have disturbed.

A block was made in which the side of the lateral incisor next the discolored canine was deeply stained with platina, and a most excellent imitation in color was produced, and the other teeth were vari-colored, grading in shade from one canine to the other. The effect was very good; destroying the conspicuousness which the discolored canine would have shown in contact with an unstained associate.

ourselves limited to two methods, viz. the English method of forming the gum of vulcanite the same as the base, and a platina plate with a continuous porcelain gum ; the continuous gum, as has been before remarked, presenting advantages in an artistic point of view, which are thus far unequalled.

Some effort has been made by the manufacturers to furnish an imitation of the continuous porcelain gum in a form adapted to a plastic base, and considering all the mechanical difficulties to be overcome, their efforts have resulted in considerable success.

Far more artistic talent as well as mechanical skill is required in making from a mould, a block of several teeth joined by a gum, than in the production of single teeth. The suggestions heretofore made as to their arrangement, applying here with the same force to the manufacturer as to the dentist in his adaptations to a special case. Many of the sections made for vulcanite, show conclusively that the artist who modeled them, could never have studied nature very long, nor very closely.

There is often displayed far more of the artist's invention, than his imitation. Many of the little details which go far to influence the appearance of the whole, are often neglected.

For instance, the teeth will often be fused together with particles of the tooth body left between them before baking, or what is equally common to find the beauty of the tooth in its form, ruined by a V shaped separation between them, terminating in contact with the gum, half way up the tooth ; or, again to find the central and lateral with such a space on one side and the corresponding space filled up. The point of gum between the teeth is often pale and indistinct. In these blocks the individuality of the tooth should be especially clear ; brought out by a clean and well defined space and the color of the gum between, in sharp contrast, or the *tout ensemble* will betray the porcelain character of the material used.

The writer was shown within the past year, some blocks made by Dr. Eccleston of Oxford, N. Y., which possessed much merit. They were evidently in their form and arrangement a copy rather than an imitation of nature, and the misfortune was, that nature in her most pleasing developements had not been chosen. However they evinced earnest inquiry in the right direction. The great difficulty with all sections made in moulds is that the one set form which the mould produces is necessarily of limited adoption.

When we consider the infinite variety of the human countenance, and the equally infinite diversity in form of the jaws, which a dentist sees; (no two being exactly alike,) and then consider that there are thousands with a conformation of jaw peculiar to each, who are wearing artificial teeth of exactly the same size, shape and color, in fact all cast in the same mould, and really belonging to but one individual, we begin to realize the paucity of our resources.

**(To be Continued.)**

---

